# Tackling Heterogeneous Color Registration: Binning Color Sensors

**DOI:** 10.3390/s21092950

**Published:** 2021-04-22

**Authors:** Paul Myland, Sebastian Babilon, Tran Quoc Khanh

**Affiliations:** 1 Laboratory of Lighting Technology, Technical University of Darmstadt, Hochschulstr. 4a, 64289 Darmstadt, Germany; babilon@lichttechnik.tu-darmstadt.de (S.B.); khanh@lichttechnik.tu-darmstadt.de (T.Q.K.); 2 Department of Population Health Science and Policy, Light and Health Research Center, Icahn School of Medicine at Mount Sinai, One Gustave L. Levy Place, New York, NY 10029, USA

**Keywords:** color sensing, indoor lighting, nano-optical interference filters, color correction, color sensor binning

## Abstract

Intelligent systems for interior lighting strive to balance economical, ecological, and health-related needs. For this purpose, they rely on sensors to assess and respond to the current room conditions. With an augmented demand for more dedicated control, the number of sensors used in parallel increases considerably. In this context, the present work focuses on optical sensors with three spectral channels used to capture color-related information of the illumination conditions such as their chromaticities and correlated color temperatures. One major drawback of these devices, in particular with regard to intelligent lighting control, is that even same-type color sensors show production related differences in their color registration. Standard methods for color correction are either impractical for large-scale production or they result in large colorimetric errors. Therefore, this article shows the feasibility of a novel sensor binning approach using the sensor responses to a single white light source for cluster assignment. A cluster specific color correction is shown to significantly reduce the registered color differences for a selection of test stimuli to values in the range of 0.003–0.008 $\Delta u^{\prime }v^{\prime }$, which enables the wide use of such sensors in practice and, at the same time, requires minimal additional effort in sensor commissioning.

## 1. Introduction

Intelligent systems for interior lighting are conceived to automatically find the optimal balance between energy consumption, legal requirements, visual performance, user comfort, and health considerations [1]. A closed-loop feedback design with input from optical sensors seems to be most expedient and has already been adopted successfully in practical research [2,3,4,5,6,7,8] in order to ensure that such systems are capable of monitoring and dynamically adapting to continuous changes in the environmental conditions and the lighting parameters, e.g., caused by variations of the natural daylight entry through windows and skylights [9,10,11] or by degradation and temperature processes in the luminaires [12,13,14].

In their simplest form, closed-loop control systems make use of an outdoor daylight sensor in combination with some additional light sensors that are attached to the luminaires and/or the occupants’ working area to measure the amount of daylight falling into the room in order to adapt the contribution of the artificial illumination accordingly. In extension of simple occupancy monitoring, recent studies have shown the great energy saving potentials that emerge from such sensor-driven daylight harvesting strategies [15,16,17,18,19]. In addition to the aspect of energy saving, ergonomic considerations in lighting are becoming increasingly important. Modern, multi-channel LED systems offer a huge flexibility in providing dynamic patterns of light exposure throughout the day [20], which can be tailored to match the users’ specific needs in terms of circadian rhythm [21,22,23], task-related performance [20,24,25], and lighting preference [26,27,28,29]. With regard to an intelligent lighting control, sensor feedback may thus allow for an automated time- and task-dependent adaptation of corresponding light levels and spectra, while taking contributions from time-varying natural daylight sources into account. However, as system complexity increases, advanced sensor technologies are required to implement such highly sophisticated spectral monitoring and feedback control functions.

Instead of using simple photodetectors, Chew et al. [2] and Maiti et al. [7], for example, applied optical color sensors with three or more distinct spectral channels to develop closed-loop light control schemes for multi-primary LED luminaires. The data that are delivered by these sensors can be used to mathematically approximate human trichromatic responses directly in the feedback loop, which allows for a more vision-related optimization. In both cases, high performance accuracy could be achieved: maximum deviations in chromaticity from time-varying daylight patterns or other target test spectra were found to fall well within a five-step MacAdam ellipse [30]. Deviations in correlated color temperatures (CCTs) and illuminance levels were always smaller than 5% of their target values. Botero-Valencia et al. [31] used low-cost RGB color sensors to classify light sources according to whether they are fluorescent, incandescent, or LED-based. Adopting a *k*-nearest neighbors approach, a high classification accuracy of more than 96% was reported for a sample of 54 different light sources commonly found in residential and commercial environments. In addition, they demonstrated the feasibility of cluster-specific regression models to be applied for the estimation of corresponding CCT and color rendition measures. In a similar work, the same authors [32] adopted linear regression to transform RGB sensor outputs to CIE tristimulus values first. The tristimulus values were then used to calculate corresponding CCTs. Errors in CCT estimation of less than 6% were reported for a selection of typical indoor light sources.

Despite these promising examples of successful system integration and analysis, large-scale applications of multi-channel color sensors for intelligent lighting control still pose some defying challenges. One is the fact that even same-type color sensors show production-related differences in the spectral sensitivities of their individual channels, independent of the manufacturer or the chosen manufacturing process. However, to the authors’ best knowledge, no systematic research has been published yet dealing with these variations in the spectral sensitivities of color sensors and their implications. In addition, studies involving the parallel use of multiple color sensors are generally sparse in the literature. Among the few that exist, the most relevant for the present work are those of Ashibe et al. [33] and Woodstock et al. [34]. Based on color sensor feedback, Ashibe et al. developed a method for luminance and chromaticity control of a lighting system comprising several luminaires that were installed in a real-sized model office. While their tests involving a single RGB sensor were successful, the system did not meet the performance expectations when signals of two spatially distributed sensors were used as the corresponding inputs. Unfortunately, no specification of the employed color correction procedure was provided. Furthermore, it was not investigated whether the problems observed when applying more than one color sensor for feedback control could have been caused by differences in the color registration between the individual sensors. In the work of Woodstock et al., an array of 53 RGB sensors distributed in the ceiling of a real-sized experimental meeting room serves as an integral part of a privacy-preserving occupant detection and tracking system that links sensor data to occupant location and related occupant color information. The test measurements for the same color of occupant clothing showed a large scatter in RGB coordinates between different sensors even after reducing the noise that is caused by light reflected from other objects in the room by adequate Kalman filtering. Again, no further discussion or analysis as to whether these deviations may have been caused by general differences in color registration was provided.

Despite the sparseness of literature on that topic and the lack of information provided by the few references that have been identified as relevant for the present work, it should have become clear that sources of errors in color registration exist, even between identical color sensors and that, if more than one sensor is used in parallel, potential differences must be corrected appropriately. This holds especially true with regard to the sketched use-case of intelligent lighting control. However, as the number of color sensors increases, proper sensor adjustment gets increasingly complex, time-consuming, and, as a consequence, impractical for the provider/operator of such systems.

In order to overcome this problem, the following paper presents the first systematic research on the differences in color registration between same-type color sensors and introduces a novel binning framework for an efficient sensor characterization, which makes use of the sensors’ channel responses to a selection of test light sources to determine similar clusters of sensor behavior. The cluster centroids are then used for a one-time adjustment process. The resulting cluster-specific transformation functions are eventually applied for correcting the output of the individual color sensors that belong to the same cluster without the need for repeating the time-consuming adjustment for each sensor. Further details on the implementation and accuracy of this new approach will be discussed in the following.

## 2. Materials and Methods

### 2.1. Sensor-Based Color Registration and Related Issues

A color sensor can be modeled according to Equation (1) [35]. The integral over the color stimulus spectrum ϕ(λ) weighted by the spectral sensitivity function $s_{k}\left(\lambda \right)$, while taking into account the exposure time *e* and the gain factor κ, results in the output value $c_{k}$ of the *k*th color sensor channel. The color stimulus function ϕ(λ) is obtained from multiplying the spectral radiance of the illumination E(λ) with the spectral refectance r(λ) of an object that is observed by the color sensor. The function *F* in Equation (1) allows for incorporating nonlinear behavior in the sensor model. The term $n_{k}$ includes additive noise that is imposed on the sensor responses.

(1) $$\begin{array}{cc} c_{k} & =F(\kappa ,e,S)\\ S & =\int \phi \left(\lambda \right)\;\cdot \;s_{k}\left(\lambda \right)\;d\lambda +n_{k}\\ & =\int r\left(\lambda \right)\;\cdot \;E\left(\lambda \right)\;\cdot \;s_{k}\left(\lambda \right)\;d\lambda +n_{k} \end{array}$$

**Figure 1:**
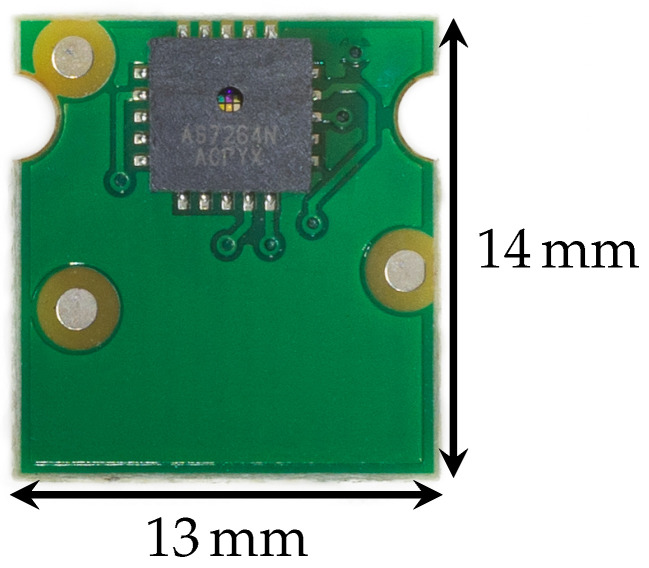
Three-channel true color sensor on a circuit board with rear contacting and milled holes for mounting. The sensor’s surface with its distinct channels can be spotted through the circular opening in the housing.

In this work, so-called true color sensors of 16 bit digital resolution will be considered. These sensors, a sample of which is illustrated in Figure 1, consist of three distinct channels whose spectral sensitivities are designed to resemble the color matching functions $\bar{x}\left(\lambda \right)$, $\bar{y}\left(\lambda \right)$, and $\bar{z}\left(\lambda \right)$ of the CIE 1931 2° standard observer in order to imitate human color perception. Nano-optical Interference filters with Gaussian transfer functions that are coated directly onto the surface of a CMOS photodetector are used to create this specific transmission behavior.

The integration times and gain factors are assumed to be fixed and equal for different sensors. For this reason, the function *F* in Equation (1) can be substituted by a simple multiplication with a constant δ. The discretized sensor model then reads

(2) $$c_{k}=\delta \;\cdot \;\sum_{}^{}\phi \left(\lambda \right)\;\cdot \;s_{k}\left(\lambda \right)\;\cdot \;\Delta \lambda +n_{k}.$$

Note that quantization errors are neglected here, as discretization-induced deviations between the model and the real sensor data are expected to be very small because of the 16 bit resolution that is offered by each channel of the considered sensor type. The integral of Equation (1) is approximated by discrete summation in steps of Δλ = 1 nm.

For a colorimetric use of the color sensor data, the raw sensor outputs of Equation (2) must be transformed to CIE XYZ tristimulus values first, in this work the 2° observer is used in the calculation. This essential step of sensor adjustment, also called color correction, is well-known from digital camera systems. Usually, assuming that the Luther–Ives condition [36,37] is met sufficiently, a linear matrix transformation of the form

(3) $$\left(\begin{array}{c} X\\ Y\\ Z \end{array}\right)=M_{3\times 3}\;\cdot \;\left(\begin{array}{c} c_{X}\\ c_{Y}\\ c_{Z} \end{array}\right)$$

is used to convert from sensor outputs to the device independent XYZ color space with as little error as possible. Thus, the transformation matrix $M_{3\times 3}$ is obtained by minimizing the corresponding mean squared error of the linear mapping, i.e.,

(4) $$M_{3\times 3}=\underset{M}{arg min}\{||XYZ-M\;\cdot \;C_{XYZ}{\left|\right|}^{2}\}.$$

In order to solve this least-squares optimization problem in a practical manner, the color sensor must be exposed to a set of color stimuli “L” whose XYZ tristimulus values are known, e.g., by measurement using a color- or spectrometer. In this work, a selection of 318 different light spectra published together with the TM-30-18 standard of the Illuminating Engineering Society (IES) for the evaluation of a light source’s color rendition [38,39] has been adopted as the corresponding set of color stimuli. The IES TM-30-18 comprises a set of standardized measures and calculation methods for an accurate quantification of the color rendition characteristics of a light source based on a model of human color vision. Basically, the appearance of a selection of color samples illuminated by the light source to be tested is compared to their appearance under a reference light source of the same CCT, which is either a Planckian radiator (CCT≤4000K), a CIE daylight phase (CCT≥5000K) or a proportional blend of these two types of illuminants (4000K<CCT<5000K). For implementation and validation purposes, a comprehensive spectral database of light sources ranging from fluorescent lamps via incandescent and high intensity discharge lamps through to various LED mixtures and phosphor-converted LEDs has been assembled and made available for download by the IES. Apart from covering a broad range of different lighting technologies for an increased general validity, using this well-defined and approved collection of light spectra to solve Equation (4) further ensures the reproducibility.

The resulting sensor outputs in combination with their associated XYZ values can eventually be used to calculate $M_{3\times 3}$. However, the linear transformation matrix determined for a specific color sensor in general cannot be transferred to another one (even of the same kind) without introducing large colorimetric errors. Consequently, the time-consuming color correction has to be repeated for each and every color sensor.

This becomes more clear when looking at the individual spectral sensitivities of different, but identical, color sensors. For the exact determination of such channel sensitivity curves, a monochromator setup is frequently used in the literature [35,40,41,42,43,44] and was also adopted here. Based on the model that is derived from Equation (1), the spectral sensitivity of a sensor channel *k* can be determined from the normalized sensor responses to a set of narrowband color stimuli $\phi_{\lambda }\left(\lambda \right)$ provided by the monochromator. The corresponding functional relationship reads

(5) $$s_{k}\left(\lambda \right)=\frac{F^{-1}\left(c_{k}\left(\phi_{\lambda }\right)\right)-n_{k}}{e\;\cdot \;\int \phi (\lambda )\;d\lambda }\approx \frac{c_{k}\left(\phi_{\lambda }\right)-{\bar{n}}_{k}}{\delta \;\cdot \;\sum \phi (\lambda )\Delta \lambda },$$

where the random noise variable $n_{k}$ is approximated by its mean value observed for a no-light condition ${\bar{n}}_{k}$ [35], i.e., when the monochromator output is closed. Minor irradiance-dependent noise contributions are neglected [45,46].

The measurements were performed on a monochromator setup using Equation (5) with a Δλ=1 nm step size. The output spectra of the monochromator showed a full width at half maximum (FWHM) of approximately 2 nm. The setup essentially consists of a six inch integrating sphere with a highly reflective Spectralon^®^ (optical PTFE) inner coating, a 300 W xenon light source in combination with a single monochromator MSH 300 (Quantum Design GmbH, Darmstadt, Germany), and a scanning spectroradiometer Spectro 320D R5 (Instrument Systems GmbH, Munich, Germany). The spectroradiometer, the monochromator output aperture, and a sensor holder are connected to the three ports of the integrating sphere, which ensures a homogeneous radiation distribution in the field of view of the color sensors.

**Figure 2:**
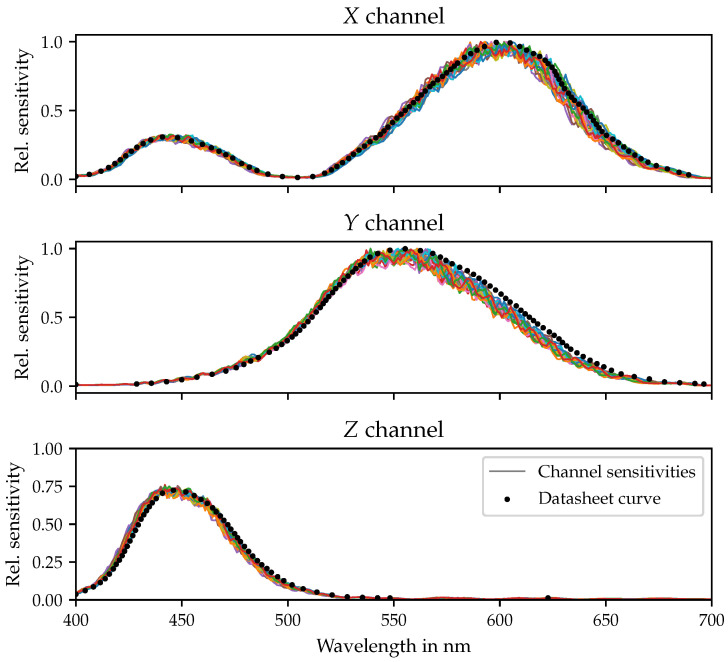
Spectral sensitivity curves (solid lines) of 24 color sensors of the same type, normalized to their sensitivity at 555 nm in the *Y* channel and compared to their corresponding data sheet values (black dotted lines).

Figure 2 shows the accordingly measured spectral sensitivity curves of the 24 same-type true color sensors that were considered in this work. Additionally plotted (dotted black lines) are the corresponding data sheet values. Pronounced deviations between individual sensors as well as between the sensors’ behavior and the data sheet curves are apparent. The largest differences between the data sheet report for the different sensor channels and the correspondingly measured sensitivity curves can be observed for the *Y* component. For the remaining channels, slightly smaller, but still considerable, deviations must be stated.

In all cases, the erratic form of the measured channel sensitivites originates from the different layers of interference filters used to approximate the CIE 2° color matching functions. The decreasing frequency of the many local maxima and minima on the sensitivity curves with increasing wavelength is typical for this technology. Layer thickness variations between the individual color sensors cause these local maxima and minima to occur at different wavelengths for different sensors and, therefore, require determining a separate color correction matrix for each color sensor.

### 2.2. New Binning Approach for Color Sensors

The individual characterization of each color sensor using a monochromator setup, as sketched in the previous section, is an expensive and time-consuming task and, therefore, highly impractical for a large-scale production. Besides the material costs and maintenance effort of a detailed characterization of optical sensors using a monochromator setup, the major disadvantage is the required time it takes to perform the measurements. Each color stimulus that is provided by the monochromator needs to be captured with both the spectroradiometer and color sensor, where adequate signal levels must be ensured by choosing sufficiently long integration times. The spectral characterization of a single sensor with a step size of 5 nm ranging from 400 nm to 700 nm (i.e., 81 measurements in total) and an assumed integration time of 700 ms (which was a typical value for the measurements reported in Figure 2) for example takes a minimum of 56.7 s without taking the times needed for preparation and the computation of the resulting spectral sensitivities into account. For this reason, the idea of the present work is to explore the possibility of classifying color sensors of the same type into characteristic bins based on their response behavior to light exposure. The subsequent determination of a single color correction matrix representing each bin is hypothesized to be sufficient for most applications, in particular for those that are related to intelligent lighting control.

To assess the feasibility of this approach, the sensor responses of the 24 true color sensors considered in this work are calculated first from the sensors’ measured spectral sensitivities for a set of probe stimuli “C”. A dedicated cluster algorithm is then applied to the sensors’ output data to define the sensor groups of equal response characteristics. The use of a single warm-white, phosphor converted LED spectrum as the respective probe is found to be sufficient for achieving an accurate classification of the sensors with regard to their channel outputs in response to that LED stimulus, as will be shown in Section 3.1.

In this work, an agglomerative cluster algorithm that is based on the Ward method [47] is applied to perform a data-driven sensor classification: starting from a single, separate cluster for each sensor, neighboring clusters are iteratively joined together, while trying to minimize their intrinsic overall variance, until the desired number *n* of sensor bins has been created. Here, the variance is measured in terms of an index based on the sum of squared distances of the sensors’ coordinates in response to the probe stimulus and the respective cluster means. A value of n=5 target clusters has been found to be sufficient for the current data sample to achieve a sensor classification into bins with approximately equidistant cluster centroids.

**Figure 3:**
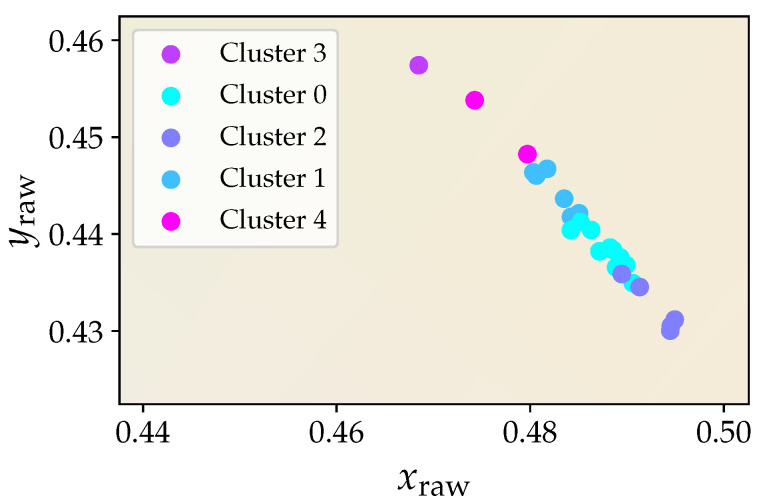
Classification of the color sensors into five bins of similar behavior in response to a warm white, phospor-converted LED spectrum. An agglomerative cluster algorithm has been applied for proper sensor assignment.

For a better visualization, Figure 3 depicts the resulting assignment of the 24 true color sensors. The shown data points represent the two-dimensional projection of the corresponding raw sensor outputs, i.e., without applying any color correction, as given by

(6) $$\begin{array}{cc} x_{\mathrm{raw},i} & =\frac{c_{X,i}}{c_{X,i}+c_{Y,i}+c_{Z,i}},\\ y_{\mathrm{raw},i} & =\frac{c_{Y,i}}{c_{X,i}+c_{Y,i}+c_{Z,i}}, \end{array}$$

where the index *i* denotes the *i*th color sensor. The cluster assignment of individual sensors that show a similar response behavior into the same characteristic bin is indicated by a respective color coding. Note that the actual clustering is performed on the sensors’ raw output data of all three channels; the two-dimensional representation of Figure 3 only serves illustrative purposes.

**Figure 4:**
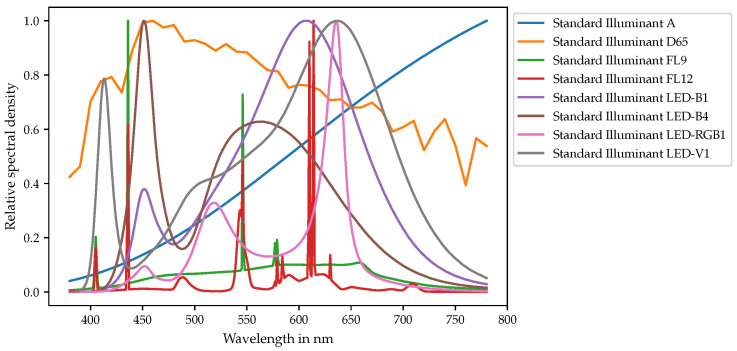
Test spectra “T” for evaluating the differences in the signals of sensors of the same type on identical illumination spectra.

Based on Equation (4), the cluster centroids are eventually used to compute cluster-specific transformation matrices. Thus, a cluster-wise color correction can be applied using the same transformation matrix for all sensors that belong to the same bin. To evaluate the accuracy of this approach and provide an adequate proof of concept, color differences for a selection of test stimuli “T” are calculated to compare the results observed for the cluster-wise color corrected sensors with those that were obtained for the CIE 2° standard observer assumed to represent the ground truth. In this work, eight different standardized CIE illuminants that are shown in Figure 4 have been chosen for this purpose, as they represent a selection of typical light spectra encountered for in- and outdoor applications.

**Figure 5:**
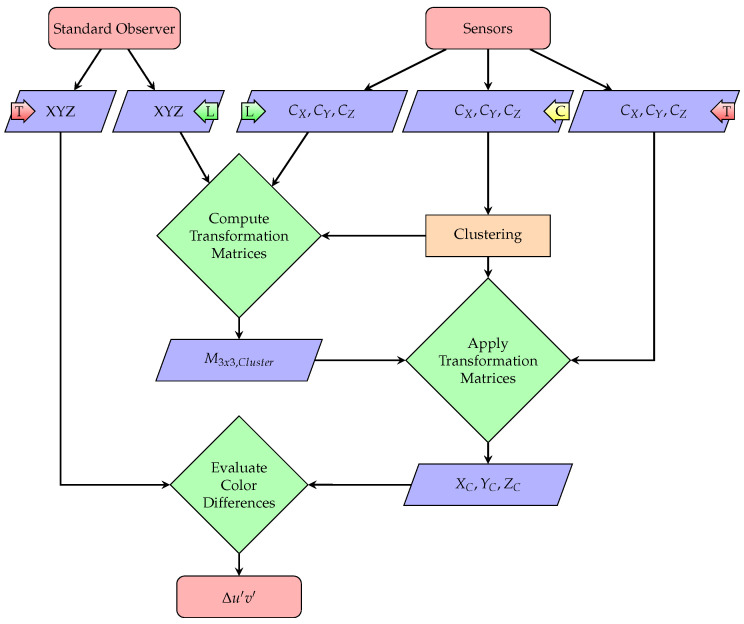
Flow chart of the cluster and evaluation procedure of the new sensor binning approach proposed in this work.

For a better overview, the whole cluster and evaluation procedure is summarized by the flow chart of Figure 5: color sensors are first clustered based on their responses to a set of probe stimuli “C”. The cluster centroids are then used to determine a single color correction matrix from a set of known color stimuli “L” that is applied to all sensors within the same bin showing a similar response behavior. The accuracy of the approach is finally evaluated by calculating the color differences between the 2° standard observer and the cluster-wise color corrected sensors for a set of test stimuli “T”. All of the chromatic calculations are performed using LuxPy [38]. The results of the proposed binning approach in terms of color correction accuracy will be discussed in the following.

## 3. Results

### 3.1. Impact of Probe Stimuli on Cluster Results

This section examines the impact of different selections of probe stimuli “C” on the accuracy of the sensor classification. From the literature dealing with observer variability [48,49], it can be expected that, due to the observed variations in the local minima and maxima modulating the sensors’ spectral sensitivities (see Figure 2), the use of narrow probe stimuli may result in larger deviations between different sensors in terms of their response behavior, which could be beneficial for the clustering process.

In Table 1, the mean, maximal, and minimal color differences $\Delta u^{\prime }v^{\prime }$ between the cluster-wise color corrected sensor responses and the corresponding color coordinates of the CIE 2° standard observer assumed to represent the ground truth for the set of test spectra “T” are summarized for different selections of probe stimuli “C”. Thus, in each case, the indicated values describe the differences between the color perceived by a standardized human observer and the sensor outputs after previous classification and cluster-wise color correction. From these data, it becomes evident that for this sample of sensors applying the cluster algorithm on the sensor responses for a single white light spectrum as the probe stimulus performs as good as or even better than using a large number of different spectra or a selection of narrowband monochromatic LEDs. Indeed, the overall best performance is observed for a single warmwhite LED spectrum, which is therefore adopted as the probe stimulus “C” for all subsequent performance evaluations.

This finding can be explained with regard to the method of color correction used. The simple 3×3 approach of Equation (4) essentially adjusts the relative gain of the individual sensor channels in relation to each other by means of a simple linear combination of their corresponding spectral sensitivities to minimize the mean squared mapping error for a set of color stimuli “L”. Thus, a white light source without pronounced gaps in its spectrum, for example a warm white LED or even a tungsten illuminant, provides sufficient information on the general deviations of the sensors’ global response behavior (and the global shape of their spectral sensitivities) to allow for a proper classification to guarantee a sufficiently accurate cluster-wise 3×3 color correction.

On the contrary, the use of narrowband or monochromatic light sources may lead to unsuitable classifications, as these spectra are not capable of probing the global trend in the sensors’ sensitivity curves but rather capture their local variations in the spectral regions of their peak wavelengths. Because of the pronounced local differences in the spectral sensitivities (Figure 2), this may perturb a proper clustering and, thus, lead to a slightly worse performance with regard to the cluster-wise color correction procedure for white spectra. As can be seen from the last row of Table 1, making cluster assignments based on the sensor responses to single red (peak at 613 nm, FWHM 15.3 nm), green (peak at 527 nm, FWHM 29.5 nm), and blue (peak at 462 nm, FWHM 17.9 nm) LED spectra results in the worst performance among all of the considered selections of probe stimuli. A one-tailed paired t-test further reveals that using the warmwhite LED spectrum for cluster assignment significantly (t = 7.5401, df = 191, *p*-value = 9.235 × 10${}^{-13}$) reduces the color differences as compared to using this selection of monochromatic LEDs. If, however, a more locally effective color correction was used, for example a look-up table, a higher-dimensional matrix, or an artificial neural network, the accentuation of local differences by narrowband spectra could enhance performance. However, this is beyond the scope of the present work. Here, we focus on the performance evaluation of the proposed sensor binning approach using a simple 3×3 transformation matrix for color correction, as this is also the method of choice applied by the majority of manufacturers and, thus, seems to be of the most practical relevance.

### 3.2. Performance Evaluation of Sensor Binning

**Figure 6:**
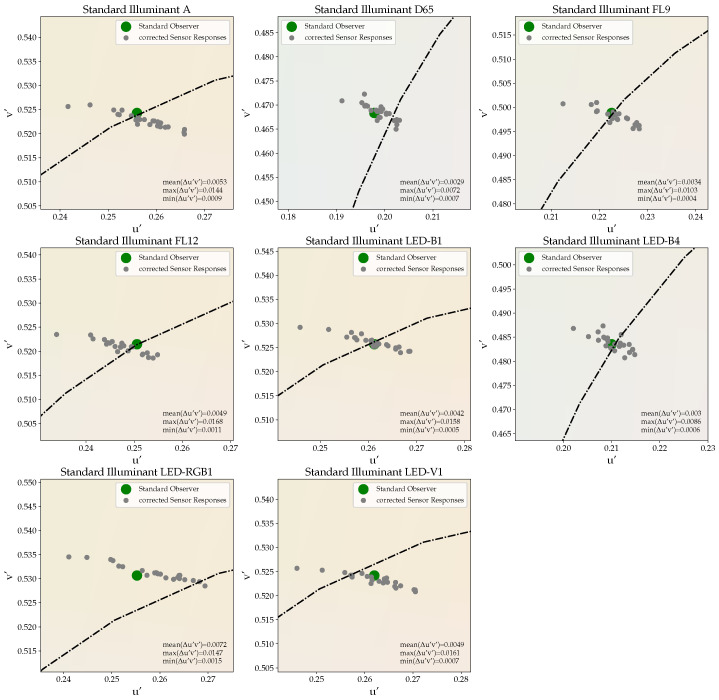
Chromaticity coordinates of the selection of test spectra “T” obtained for the 24 same-type true color sensors considered in this work. A single transformation matrix is used for the color correction of all sensors.

Figure 6 illustrates the color-corrected sensor outputs that were obtained for the different test stimuli “T” for the case that a single transformation matrix is calculated on the mean sensor outputs in response to the set of color stimuli “L”. This corresponds to the assumption that all sensors belong to the same cluster, which basically represents the current standard procedure found in the industry. The resulting XYZ tristimulus values are converted to $u^{\prime }v^{\prime }$ chromaticity space before being plotted. As can be seen, large deviations in color registration occur between the color-corrected sensor responses and the true chromaticity coordinates of the different test light spectra, as perceived by the CIE 2° standard observer. The average color difference is found to be $\Delta u^{\prime }v^{\prime }=0.0045$. However, considerably larger differences for individual sensors of up to $\Delta u^{\prime }v_{\max}^{\prime }=0.0168$ must be stated, where the largest deviations are observed along the $u^{\prime }$ axis for the present sample of true color sensors.

**Figure 7:**
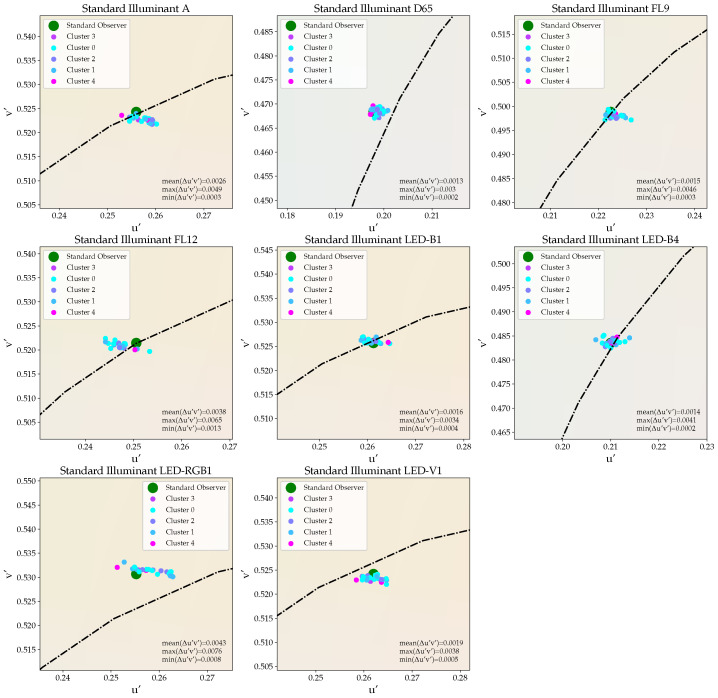
The results of a cluster-wise color correction of the 24 same-type true color sensors considered in this work after applying the proposed sensor binning approach.

If, instead, the sensors are divided into five different clusters using the proposed sensor binning approach that is discussed in Section 2.2 with a cluster-wise color correction being applied, significantly reduced deviations in color registration between the color-corrected sensor outputs and the true chromaticities of the different test spectra can be achieved, see Figure 7. The average and maximal color differences decrease to $\Delta u^{\prime }v_{\mathrm{mean}}^{\prime }=0.0023$ and $\Delta u^{\prime }v_{\max}^{\prime }=0.0076$, respectively. In addition, the dominance of the $u^{\prime }$-axis in terms of color differences, which was prevalent when using the same transformation matrix for the color correction of all sensors (Figure 6), is considerably less pronounced when applying a cluster-wise color correction.

A statistical analysis is performed to validate the results. A two-way repeated-measures ANOVA is computed for this purpose. All of the effects are reported as significant at *p* < 0.05. There was a significant main effect of the correction scheme on the resulting color differences, p<0.0001, ϵ=0.176, F(1,23)=14.474. Mauchly’s test indicated that the assumption of sphericity had been violated for the main effect of test spectrum, W=1.131 × 10${}^{-3}$, p<0.0001, and the interaction effect of correction scheme and test spectrum, W=5.61 × 10${}^{-5}$, p<0.0001. Therefore, degrees of freedom were corrected using Greenhouse–Geisser estimates of sphericity for the main effect of test spectrum, F(3,69)=22.672, p<0.0001, ϵ=0.2, and the interaction effect of correction scheme and test spectrum, F(2.44,56.1)=4.099, p<0.02, ϵ=0.019, which were both found to be significant. Subsequent Bonferroni post-hoc testing revealed that for the test spectra A (p=0.000886), D65 (p=0.000872), FL9 (p=0.002), LED-B1 (p=0.003), LED-B4 (p=0.000918), LED-RGB1 (p=0.001) and LED-V1 (p=0.000475) there were significant differences between mean-matrix and cluster-wise color correction. Only for the test spectrum FL12, no significant differences could be reported between the different correction schemes.

## 4. Discussion

Using several color sensors in parallel is not as straight forward as one might expect. From Figure 6, it is clear that large deviations in color registration from ground truth and between sensors may occur, even for theoretically identical color sensors, which still prevents their large-scale use for intelligent lighting control, as sketched in the introduction of this article. Applying a standard procedure of color correction, maximal deviations from ground truth of up to $\Delta u^{\prime }v_{\max}^{\prime }=0.0168$ have been observed, with an even larger maximal spread between the sensors. These deviations are mainly due to the erratic form of the sensors’ spectral sensitivities showing significant differences between their local minima and maxima, as shown in Figure 2.

Applying the proposed binning approach, which is based on the sensors’ channel outputs in response to a set of probe stimuli, characteristic sensor clusters have been defined. By adopting a cluster-wise color correction, the registered color differences for the selection of test stimuli could be limited to values in the range of 0.003–0.008 $\Delta u^{\prime }v^{\prime }$, which is approximately a factor of two smaller than the deviations obtained for the standard color correction procedure and corresponds with the binning specifications standardized for white LEDs. This improvement in color registration was confirmed to be significant by appropriate statistical analysis. A more fine-tuned sensor classification with more than five target clusters may further improve the color correction performance. However, their proper definition demand for a much larger number of identical color sensors to be considered than the 24 sensors that are available for the current proof of concept.

With regard to the practical feasibility of the proposed binning approach, it could be shown that a single white light source without pronounced gaps in its spectrum serving as the probe stimulus is sufficient for achieving an accurate classification of the color sensors from their corresponding channel responses. For the present sample of sensors, the best performance was achieved using a warm-white, phosphor-converted LED spectrum. This finding is very promising in terms of future standardization and integration ambitions for large-scale productions. Single LED-based probing solutions may be relatively easily implemented into existing manufacturing processes because of their compact design, low power consumption, and outstanding long-term stability.

In this context and with regard to a potential future standardization, the proposed binning approach must be extended by a pre-definition of fixed characteristic clusters, like e.g., the chromaticity quadrangles of the ANSI binning standard for white LEDs. Instead of creating for each new sample of identical color sensors a new cluster assignment, the use of such pre-defined clusters allows for a generic sensor classification, which, as described above, can be assessed by a simple one-time measurement. A corresponding partitioning of the sensors’ raw output space similar to the ANSI binning standard seems to be reasonable. However, for this purpose, again a much larger number of same-type color sensors than the 24 units considered as part of this proof of concept will be needed to increase generalizability to a necessary degree. Furthermore, it is important to figure out whether the present concept can be transferred to other color sensor technologies that are not based on nano-optical interference filters.

So far, the present work showed the feasibility and discussed the merit of a new sensor binning approach that provides a fundamental contribution towards the large-scale application of multi-channel color sensors for intelligent lighting control. In addition to this very specific field of application, future research should also focus on a potential extension of the approach beyond the sensing of color, such as e.g., for the sensor-based estimation of photosynthetically active radiation (PAR) in horticultural lighting or the acquisition of other photometric quantities.

## 5. Conclusions

In this work, a new binning approach for color sensors has been proposed. Discussing their potential use as an integral part of a large-scale implementation of intelligent lighting systems with several of these sensors working in parallel, it has been shown that considerable deviations in color registration may occur, even between same-type color sensors. An adequate correction of these errors is required in order to ensure reliable feedback for system control. However, the standard procedures of color correction have proven to be either impractical in application (e.g., when trying to determine a separate color correction matrix for each color sensor) or still lead to large deviations from ground truth (e.g., when using a single transformation matrix based on data sheet curves or determined from the means of a limited number of sensor measurements). To overcome these problems, it has been shown that the sensors’ channel outputs in response to a set of probe stimuli can be used to define characteristic sensor clusters. Furthermore, it has been demonstrated that an accurate classification can be achieved by using a single warm-white, phosphor-converted LED spectrum to serve as the corresponding probe stimulus. By performing a cluster-wise color correction that was determined from the respective cluster centroids, the differences in color registration between individual sensors as well as their deviations from ground truth can be reduced significantly. The corresponding results fall within the range of the binning specifications standardized for white LEDs.

The current work provides a significant contribution towards a similar standardization for the binning of color sensors. Tackling the problem of heterogeneous color registration, the proposed binning procedure to the authors’ best knowledge is the first approach ever that provides a dedicated framework for an adequate classification of color sensors complying with their responses to light exposure. Thus, the present work, implying future developments towards standardization, is of high practical relevance not only for the field of lighting, but also for a wide range of applications that are related to industrial processing [51], where color sensors are either used for actual sensing and color discrimination purposes or as an integral component for system control [52,53,54,55]. Further application examples of color sensors extend to diverse areas, such as medical diagnosis [56,57,58], health monitoring [59,60], biochemical sensing [61,62], plant recognition in agriculture [63], the color management of consumer electronics [64,65,66], and many more. In all these cases, knowing about the sensor characteristics in response to light exposure is crucial for proper system behavior, emphasizing the practical importance of the present work.

## Figures and Tables

**Table 1:** Resulting color differences after cluster-wise color correction for different selections of probe spectra used for determining cluster assignments.

Probe Stimuli	Source	#	Mean $\Delta u^{\prime }v^{\prime }$	Max $\Delta u^{\prime }v^{\prime }$	Min $\Delta u^{\prime }v^{\prime }$
Warmwhite	own measurement	1	0.0023	0.0076	0.0002
Coldwhite	own measurement	1	0.0027875	0.0096	0.0001
TM-30-18	[38,39]	318	0.002375	0.0092	0.0001
Illuminant A	CIE A [38,50]	1	0.0024375	0.0081	0.0001
RGB Mix	CIE RGB1 [38,50]	1	0.0025625	0.0093	0.0001
Fluorescence	CIE FL12 [38,50]	1	0.0024625	0.0115	0.0001
R,G,B	own measurements	3	0.0035125	0.0128	0.0001

## Data Availability

All data generated or analyzed to support the findings of the present study are included this article. The raw data can be obtained from the authors, upon reasonable request.

## Abbreviations

The following abbreviations are used in this manuscript:

LED	Light-emitting diode
CCT	Correlated color temperature
CIE	Commission Internationale de l’Éclairage
CMOS	Complementary metal-oxide-semiconductor
IES	Illuminating Engineering Society
TM	Technical memorandum
FWHM	Full width at half maximum
PTFE	Polytetrafluoroethylene
ANOVA	Analysis of variance
ANSI	American National Standards Institute
PAR	Photosynthetically active radiation
